# Placebo in Paediatric Clinical Trials: Systematic Literature Review and Framework‐Based Synthesis

**DOI:** 10.1111/jpc.70399

**Published:** 2026-04-27

**Authors:** Georgia Parry, Artur Abelian, Chloe C. E. Williams, Lydia E. Roberts, Megan Athersmith, Louise Oni

**Affiliations:** ^1^ Department of Paediatrics Maelor Hospital Wrexham UK; ^2^ Department of Paediatric Nephrology Alder Hey Children's Hospital Liverpool UK; ^3^ Department of Women's and Children's Health, Institute of Life Course and Medical Sciences University of Liverpool Liverpool UK; ^4^ Department of Cardiovascular Sciences University of Leicester Leicester UK; ^5^ Department of Paediatrics University of Leicester NHS Trust Leicester UK; ^6^ Department of Paediatric Nephrology Great Ormond Street Hospital for Children's NHS Foundation Trust Hospital London UK; ^7^ Department of Renal Medicine University College London London UK

**Keywords:** clinical trials as topic, ethics, paediatrics, placebos

## Abstract

**Introduction:**

Placebo‐controlled trials can provide strong evidence on safety and efficacy, but their ethical acceptability in paediatric research is contested because children have limited decisional capacity and placebo use may entail withholding effective therapy.

**Objective:**

To identify the ethical and methodological conditions under which placebo controls are acceptable in paediatric clinical trials, using a systematic review of the literature and regulatory guidance.

**Methods:**

We searched Medline, Scopus and Embase for sources addressing the ethics, acceptability or regulation of placebo use in paediatric trials. We extracted study characteristics and ethical arguments, mapped them to beneficence, non‐maleficence, autonomy and justice, and then synthesised findings using Emanuel, Wendler and Grady's seven requirements for ethical clinical research. We also obtained expert guidance from UK, USA and European regulators.

**Results:**

We included 51 sources spanning neonatal research, disease‐specific paediatric contexts, parental perspectives and general ethics/regulatory discussions. Placebo was generally considered acceptable when methodologically necessary and scientifically sound, when no proven effective therapy was withheld (or any withholding carried minimal incremental risk), and when safeguards such as rescue therapy, early escape, and close monitoring were in place. Common emphases included limits on non‐beneficial risk in children, assent and parental permission, and fair participant selection.

**Conclusion:**

Placebo use in paediatric trials is acceptable only under constrained conditions centred on scientific necessity, minimising harm from withholding therapy, robust safeguards and appropriate permission/assent processes. Further work should translate these conditions into disease‐specific, operational guidance.

## Key Points

- Placebo use in paediatric trials may be acceptable in clinical equipoise, lack of proven therapy and with robust safeguards, permission/assent and minimal incremental risks.
- There is a consensus between the literature and regulatory bodies regarding the acceptability of placebo use in paediatric trials.
- Future work should capture the views of parents, children, and clinicians and generate guidance for specific diseases.

## 1 Introduction

Clinical trials are essential for advancing medical knowledge. Placebo‐controlled trials can provide strong evidence on efficacy and safety, particularly when outcomes are subjective, disease course is variable, or adverse events are difficult to distinguish from symptoms of the underlying condition. A placebo is commonly defined as an inert substance or procedure used as a control in interventional clinical trials [1]. In paediatrics, placebo use is frequently debated because children require additional protections and placebo arms may entail delaying or withholding effective therapy [2, 3].

Paediatric evidence gaps remain clinically important: many medicines used in children lack robust paediatric‐specific efficacy, dosing and safety data, contributing to off‐label prescribing. Although children and adults share many therapeutic needs, extrapolating evidence is not uniformly straightforward. For many conditions and outcomes adult data may be informative, but uncertainty can persist regarding dose–response relationships, safety signals or across developmental stages. Differences may be substantial in some age groups (especially neonates and young infants) and for drugs with narrow therapeutic indices or developmentally sensitive pharmacological properties [4, 5]. Where uncertainty remains, reliance on adult evidence alone may risk therapeutic inadequacy, toxicity or adverse impacts on relevant childhood outcomes, including growth and development [5].

In paediatric studies there are moral, ethical and legal complexities that are less prominent in adult studies [6, 7]. Recruitment to paediatric placebo‐controlled trials can be challenging because clinicians have duties to act in the child's best interests while research aims to generate generalisable knowledge. International guidance permits placebo use where there are “compelling and scientifically sound methodological reasons” and participants are not exposed to additional risks of serious or irreversible harm through not receiving the best proven medication [2, 8]. Clinical equipoise in paediatrics can be difficult to characterise: robust adult evidence may reduce uncertainty in some circumstances, yet genuine uncertainty may persist where disease mechanisms, expected response, endpoints or safety profiles differ across ages. In these circumstances, the degree of similarity and the expected response to treatment across ages becomes central to decisions about trial design and the need for placebo controls [9, 10, 11, 12].

Further practical challenges include parental understanding of placebo and randomisation in a population that cannot provide full legal consent, alongside the developing capacity of children to provide assent or dissent [13, 14, 15]. Paediatric placebo‐controlled trials are therefore shaped by methodological justification, constraints on risk and safeguards such as rescue therapy, early escape and close monitoring, within the regulatory frameworks designed to protect children while enabling generation of high quality evidence [2, 16].

### 1.1 Objective

To identify the ethical and methodological conditions under which placebo controls are acceptable in paediatric clinical trials, using a systematic review of the literature and regulatory guidance.

## 2 Methodology

The literature review methodology was designed based on the PICO (population, intervention, comparator, outcome) format.

### 2.1 Study Population

The study population relevant to this project was any literature capturing children under the age of 18 years and focused on the theme of placebo‐controlled clinical trials. The inclusion and exclusion criteria can be found in Table 1.

**TABLE 1:** The inclusion and exclusion criteria used for the systematic literature review.

Inclusion criteria	Exclusion criteria
Discusses the ethics or acceptability of placebo use in clinical trials involving children.	Randomised controlled trials
Published between 2000 and 2025	Non‐human studies
Full text available in English	Full text not available or not in English

### 2.2 Intervention and Comparator

The intervention relevant to this systematic review was the use of placebo, compared with any intervention as a comparator in paediatric clinical trial designs.

### 2.3 Outcome

The outcome of the search was to identify the implications and circumstances surrounding the use of placebo agents in paediatric trials.

### 2.4 Study Design

#### 2.4.1 Search Strategy for Article Identification

The search strategy for the systematic review involved the use of three primary databases: Medline, Scopus and Embase. The key search terms included: ‘neonat*’, ‘infan*’, ‘child*’, ‘adolescen*’, ‘paediatric*’, ‘paediatric*’, ‘placebo*’ and ‘ethic*’. These articles were accessible through the John Spalding Library, Betsi Cadwaladr University Health Board, North Wales. EndNote Basic was used for reference management, and Rayyan software [17] supported the systematic screening process, involving four reviewers. Discrepancies were resolved through discussion.

#### 2.4.2 Data Collection

The relevant information was mapped to the four principles of bioethics [18] and synthesised using Emanuel, Wendel and Grady's clinical research ethics framework [19]. This approach maintained fidelity to the ethical arguments expressed in the sources and structured the synthesis on research ethics requirements.

#### 2.4.3 Critical Appraisal of the Included Articles

Using the applicable critical appraisal skills programme (CASP) tools, articles were scored and the results were descriptively reported. Opinion pieces, expert consensus and editorials were excluded from formal critical appraisal.

#### 2.4.4 Enquiry to Regulatory Bodies

In addition to the literature, as part of the information gathering process for this systematic review, expert opinion was obtained from the Medicines and Healthcare products Regulatory Agency (MHRA) in the United Kingdom, the FDA in the United States and the European Medicines Agency (EMA) in Europe who were contacted in November 2023, either via email or an online professional forum, to enquire about guidance for use of placebo in paediatric clinical trials. Any information that was publicly available on their websites was also viewed.

### 2.5 Ethical Approval

According to National Health Service requirements, ethical approval was not required for this systematic review because it involved a secondary review of published literature.

## 3 Results

### 3.1 Data Extraction

**FIGURE 1:**
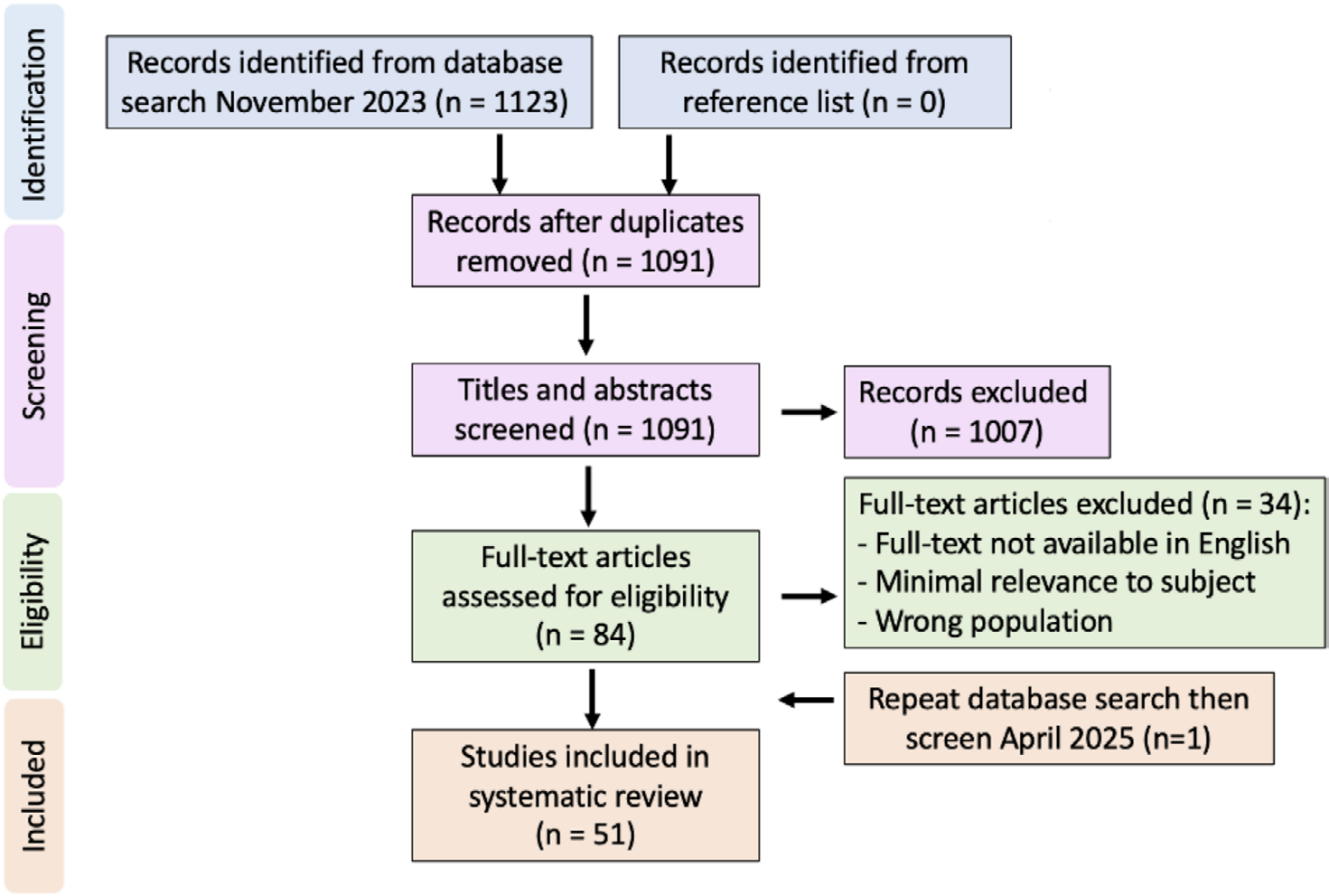
Flowchart representing the screening process in accordance with the Preferred Reporting Items for Systematic Reviews and Meta‐Analyses (PRISMA).

A total of 1123 articles were identified from a combined search of Medline, Scopus and Embase that was initially performed in November 2023 and updated in April 2025. After removing duplicates, 1091 articles remained. Independent title and abstract screening (GP and LR) yielded 84 remaining articles, and these were screened by 3 reviewers (GP, CW and AA), resulting in 50 articles. The updated search performed in 2025, using the same databases, acquired 1 further article, meaning a total of 51 manuscripts were included in the systematic review (Figure 1). The reference list of included studies did not identify any further relevant studies for inclusion.

### 3.2 Article Summary

The included articles originated from America (United States, Canada, Chile), Europe (United Kingdom, France, Germany, Finland, Italy, Switzerland, Sweden, Spain, Belgium, Slovenia, Netherlands, Ireland), Oceania (Australia, New Zealand), Africa (Ghana, South Africa), and Asia (Singapore). Overall, the articles consisted of reviews, opinion pieces, workshop and case discussions, as well as ethical analyses; they summarised professional and parental perspectives, ethical frameworks and regulatory guidance. Overall the article type was low quality evidence and there were 10 (20%) of articles suitable for formal critical appraisal. When appraised, the 3 qualitative studies scored a mean of 9.3 (range 9–10) and the 7 systematic reviews scored a mean of 7.5 (range 5–8).

There were 10 (20%) articles on the use of placebos in neonatal research (especially pain management in neonates). A further 23 (45%) articles explored specific paediatric conditions or circumstances, such as psychopharmacology [6], neurology [4], inflammatory bowel disease [3], vaccination [3], paediatric analgesia [3], urology [1], asthma [1], hypertension [1], and migraine [1]. There were 2 (4%) articles which focused on parental perspectives. The remaining 16 (32%) articles were summaries written by professionals, discussing general methodology and trial design, ethical debates regarding risks and harm of using placebo treatment, and regulatory frameworks. A comprehensive summary of the included neonatal and paediatric studies is shown in Tables S1 and S2.

### 3.3 Overview of Study Findings

Ethical issues were extracted according to beneficence, non‐maleficence, autonomy and justice (Table S1), and reported using Emanuel, Wendler and Grady's seven requirements as the primary synthesis structure [19].

#### 3.3.1 Social or Scientific Value

Social and scientific value was discussed in relation to avoiding ‘therapeutic orphanhood’ and reducing reliance on off‐label prescribing in children [20, 21, 22]. Several sources argued that neonates and children should not receive care based on weaker evidence than adults and that paediatric populations should share equitably in the benefits of scientific and medical progress [23, 24, 25, 26]. Historical drug disasters were cited as illustrating the consequences of inadequate paediatric evidence and the importance of robust paediatric trials [27]. Other sources highlighted limited paediatric efficacy data and extrapolation from adult trials [20, 21, 28]. In the principlism mapping, these arguments were most often framed as beneficence (future patient benefit) and justice (avoiding inequitable evidence standards for children).

#### 3.3.2 Scientific Validity

Across the included sources, placebo use was most frequently justified on the grounds of scientific validity, especially where outcomes were subjective, disease course variable, placebo response rates were high, or the effectiveness of standard care was uncertain. Higher placebo responses in migraine, psychiatric and neurodevelopmental contexts were described to distinguish true pharmacological effects from subjective improvement [26, 29, 30, 31]. Several psychopharmacology sources emphasised placebo response and outcome subjectivity as factors supporting placebo‐controlled designs [2, 3, 8, 20, 32, 33]. Some sources reported similarity to adult psychiatric conditions (e.g., schizophrenia and depression) when discussing active‐control designs on this topic [2, 34]. A small number of sources gave examples of how paediatric specific studies had identified important safety signals, including concerns relating to suicidality associated with SSRIs in adolescents [35]. Where authors discussed the boundaries to limit placebo use, most did not define “proven effective therapy” explicitly; with one source using the term ‘commonly accepted therapy’ [8]. One paper highlighted interpretative and ethical concerns where placebo use would require withdrawing or withholding a treatment, suggesting that this may complicate trial justification [36]. Methodological justifications were typically framed in terms of beneficence in the principlism mapping, on the basis that valid and interpretable evidence justified participation.

#### 3.3.3 Fair Participant Selection

Fair participant selection depends on factors such as disease severity, availability of effective treatment and the wider public health context. Many sources discussed that the benefits and burdens of research should be fairly distributed across society and that excluding children delays evidence for paediatric therapies [6, 23]. Some sources suggested specific inclusion criteria to protect children in certain groups (e.g., those at higher risk of disease progression and/or the most severe form of the condition) from being overrepresented [5, 8]. This could include, for example, restricting placebo use in paediatric hypertension trials to mild cases with monitoring protocols [5]. Subjecting children to placebo who are unable to provide informed consent was raised as a repeated concern, especially in the neonatal population where assent is never questionable [21, 24, 37, 38, 39].

A published literature review highlighted that 75% of studies (*n* = 98) had subjected neonates to pain [40]. It was also noted that a placebo is not ethical in severe or life‐threatening conditions if it would involve withholding effective treatment [20, 33, 41, 42]. Another study highlighted the potential public health impact, where placebo use could prevent controlling an outbreak in global emergencies, especially if there was adult data [43]. This article also emphasised inclusion of children of all ages and demographics across the world for representative evidence otherwise this may be deemed unethical [44].

A key finding was that the ethical thresholds for placebo use also differed between specialties, with several papers offering condition‐specific guidance [45]. In a study evaluating inflammatory bowel disease (IBD) trials, the authors concluded that an active controlled (ACT) trial may be more appropriate during later phase studies. They also highlighted the importance of extrapolating adult data [5, 46]. In the principlism mapping, this domain aligned mostly with justice, particularly regarding vulnerability, fair distribution of burdens and benefits, and inclusion across developmental ages and populations.

#### 3.3.4 Favourable Risk–Benefit Ratio

Across sources, discussion of the risk‐benefits clustered into recurring themes (Table 2). Many sources stated that placebo use was acceptable when no proven effective therapy exists, clinical uncertainty persists, and any incremental risk associated with placebo exposure is minimal and mitigated [2, 3, 8, 20, 32, 33, 45, 47]. Several sources noted that predicting minimal incremental risk can be challenging, particularly in populations with few prior studies [13, 33]. Condition‐specific parameters were sometimes described, for example in paediatric hypertension where placebo use was considered in mild‐to‐moderate primary hypertension in the absence of target organ damage, for short duration (e.g., 4–8 weeks), and with close monitoring [5]. In vaccine contexts, one source described a strategy to preserve allocation concealment and avoid pain by imitating vaccine delivery [48]. In prinicplism mapping, these considerations were most often framed as non‐maleficence (avoiding harm from withholding effective therapy) balanced against beneficence (values of evidence and potential participant benefit).

**TABLE 2:** Extracted key benefits versus risks of using a placebo in paediatric clinical trials.

Key benefits	Key risks
Improved interpretability of efficacy and safety by accounting for placebo effect [8, 21]. Potentially fewer participants exposed to investigational therapies compared with other trial designs [2, 46]. Paediatric specific evidence generation, reducing reliance on extrapolation and off label use [33].	Withholding treatment in severe conditions when adult data exists or when a therapy already exists (especially analgesia) may subject children to avoidable harm [20, 33, 41]. Challenging to assign duration of placebo‐controlled trial to assess long‐term implications of new therapy [34]. Potential for deterioration during placebo exposure, emphasis on rescue/escape and monitoring [8, 33, 46].

#### 3.3.5 Independent Review

The regulatory and approval measures in place to protect children were also commonly referenced in the literature. There were over fifteen different regulations or organisations mentioned across the 51 included papers [8]. The most commonly cited framework was the Declaration of Helsinki [2, 20, 27, 34, 40, 49]. In the principlism mapping, independent oversight and governance safeguards were most often framed as justice (procedural fairness) and non‐maleficence (risk constraint).

#### 3.3.6 Informed Permission and Assent

Many sources treated parental permission and child assent/dissent as central to the acceptability of placebo use, with additional concerns raised regarding placebo trials in neonates and young infants [41, 50, 51, 52]. However, some sources differentiated between types of interventions: a placebo‐controlled trial comparing formula milks compared to a placebo‐controlled vaccine study involving an intramuscular injection [2, 24, 33]. An important recurring theme was the ability to consent which changed with maturity over childhood. A few articles suggested seeking assent from around the age of 7 years, and described an understanding similar to adults by 15 years old [27, 46, 53]. In a survey of 100 paediatric researchers (66% doctors), respondents reported that children as young as 10 years old (IQR 7–12) might be capable of assent depending on the study, with full consent considered appropriate from around 14 years (IQR 12–16) [54]. One paper highlighted that adolescents could be considered for inclusion in adult trials [46]. A small number of sources cautioned that despite assent, children may remain vulnerable to coercion [21, 23]. In the principlism mapping, these arguments fell primarily under autonomy, operationalised as parental permission, age‐appropriate assent and respect for dissent.

#### 3.3.7 Respect for Potential and Enrolled Participants

Respect for participant welfare and rights was reflected through discussions of withdrawal rights, rescue obligations, ongoing monitoring, open‐label extensions, post‐trial access to effective therapies and efforts to minimise participant burden throughout the research process [3, 5, 37, 46, 55]. Specifically, add‐on and response‐adaptive designs to reduce exposure to inferior treatments were mentioned [36]. The authors in a global expert group for IBD commented that adolescents may join adult placebo trials if standard treatments have failed and subgroup size would allow meaningful analysis [56]. Several sources also emphasised large‐scale collaboration and coordination to reduce duplication of studies [20, 27, 57]. In principlism mapping, ongoing obligations (including withdrawal rights, monitoring and minimising burden) most commonly aligned with autonomy and non‐maleficence.

### 3.4 Information Gathered From Regulatory Bodies

Following an enquiry to the MHRA, EMA and FDA about guidance for use of the placebo in paediatric clinical trials, the MHRA advised that they have not published formal guidance specifically on the use of placebo in clinical trials involving children; however, they emphasised the importance of assessing each trial on an individual basis. The EMA signposted to their guidance ‘Ethical Considerations for Clinical Trials on Medicinal Products Conducted with Minors’ [58]. This document highlights that the use of placebo must be scientifically justified and the situation must have clinical equipoise. It may be used in situations where placebo effects are highly variable or in situations where alternative effective treatment is lacking. However, this guidance forbids withholding effective treatment from children, especially in serious or life‐threatening conditions. It also highlighted the problems of using a placebo intervention beyond 3–6 months and suggested that an active control arm (with or without placebo in addition) may be a suitable alternative in some circumstances. The FDA responded to signpost to their document titled ‘Ethical Considerations for Clinical Investigations of Medical Products Involving Children’ [56]. This document highlights that sometimes placebo‐controlled trials are needed in the childhood population; however, there must be a scientific necessity and there must not be a ‘minor increase over minimal risk’ without the prospect of direct benefit for the child. They highlighted the importance of review by an Institutional Review Board (IRB) and parental informed consent with assent from the child (where appropriate).

## 4 Discussion

This systematic review synthesised academic and regulatory sources on the ethical acceptability of placebo use in paediatric clinical trials. By mapping arguments to the Beauchamp and Childress' four principles of bioethics and synthesising them using Emanuel, Wendler and Grady's seven requirements, we reviewed methodological justification, risk constraints and procedural safeguards in a way that is directly relevant to research design.

The ethical acceptability of placebo in paediatric trials is best understood as a decision problem: balancing the need for reliable, interpretable evidence against limits on incremental harm from withholding effective therapy, implemented through robust safeguards, meaningful parental permission/assent, fair recruitment and independent oversight. In the included literature, the use of a placebo was defended where it was considered methodologically necessary to distinguish true treatment effects from non‐specific improvement (particularly with subjective outcomes, variable disease course, or high placebo response), and challenged where it would entail withholding effective therapy, particularly in severe, painful, or life‐threatening conditions.

More specifically, when the following criteria are met: (1) the research question has clear social values for children [13, 21, 41] and is scientifically justified [21, 23, 49, 59, 60], (2) incremental risks are minimal or mitigated [2, 3, 8, 20, 32, 33], (3) safeguards are embedded in the protocol and adequately scrutinised [2, 16, 20, 27, 34, 40, 49], (4) permission/assent processes are developmentally age‐appropriate [2, 27, 33, 46, 53] and (5) participant selection is fair [8, 23]. These extracted key considerations are broadly consistent with the regulatory guidance [38, 61, 62, 63, 64].

A challenge to applying these conditions is the subjective definition of ‘proven effective therapy’. Many sources referenced this concept without defining it, and, as a result, determining whether placebo entails withholding an effective intervention requires careful judgement for the disease and situation. The review also highlights that placebo ethics in paediatrics is not uniform across age groups [2, 27, 33, 46, 53]. Neonatal contexts were frequently discussed in relation to pain and the absence of assent [65].

This study does have limitations in that the evidence used to compile the thematic analysis was low grade. Notably, empirical evidence on stakeholder perspectives was limited, with relatively few studies examining parents' or children's views, indicating an important area for future work [8, 32, 66]. This review is limited by the heterogeneity of included sources and by representation of perspectives. Consequently, the review offers general conditions for acceptability; however, further work would need to determine disease‐specific recommendations [67].

## 5 Conclusions

Taken together, the acceptability of placebo use in paediatric trials is complex. Future work should translate these general conditions into operational, disease‐specific guidance incorporating stakeholder perspectives, with explicit definitions relevant to the disease area, to outline the recommended placebo duration in a master trial protocol design that could incorporate pre‐specified rescue or escape thresholds.

## Funding

The authors have nothing to report.

## Conflicts of Interest

The authors declare no conflicts of interest.

## Supporting information


**Table S1:** A summary table of the included neonatal studies and the key themes raised according to the four principles of clinical ethics.
**Table S2:** A summary table of the included paediatric studies and the key themes raised according to the four principles of clinical ethics.

## Data Availability

The data that supports the findings of this study are available in the Supporting Information of this article.
