# Supplementary material for: Placebo in Paediatric Clinical Trials: Systematic Literature Review and Framework‐Based Synthesis
Source: J Paediatr Child Health. 2026 Apr 27;62(6):916–23. doi: 10.1111/jpc.70399 (PMC13254118; doi:10.1111/jpc.70399)
Supplement: Supplementary file 1 — Table S1: A summary table of the included neonatal studies and the key themes raised according to the four principles of clinical ethics. Table S2: A summary table of the included paediatric studies and the key themes raised according to the four principles of clinical ethics. [file JPC-62-916-s001.docx]

**Supplementary information
Table S1**: A summary table of the included neonatal studies and the key themes raised according to the 4 principles of clinical ethics.

| **Aim / Summary** | **Beneficence** | | **Maleficence** | | **Autonomy** | **Justice** |
| --- | --- | --- | --- | --- | --- | --- |
| Paper 1: Allmark et al, Is it in a neonate's best interest to enter a randomised controlled trial? (18) | | | | | | |
| Establish whether it is in a neonate’s best interest to enter into an RCT by discussing 3 hypothetical scenarios. | | Neonate may benefit from new treatment in finely balanced circumstances. | | Avoid any burdensome procedures to administer placebo (e.g. IM injection). It suggests mimicking the process of injection as an example to avoid this. | Neonates lack autonomy and are a unique group. They cannot provide a prior preference like an adult anticipated to lose capacity, nor can you rely on developing autonomy like an older child. | Neonates do not deserve to be treated on the basis of poorer quality evidence compared to adults. Neonates have a “stake” in scientific and medical progress. |
| Paper 2: Amin et al, Clinical trials of drugs used off-label in neonates: ethical issues and alternative study designs (28) | | | | | | |
| Investigate the ethical challenges in conducting RCTs for off-label therapies in neonates and recommend trial designs. | | Well-designed RCTs can clarify safety and efficacy of off-label drugs, as shown by the 2006 New England Journal of Medicine caffeine trial for apnoea of prematurity. Both immediate and long-term benefits must be weighed. | | Placebo use in critically ill neonates risks withholding life-saving treatment. Add-on designs and pre-clinical studies can help mitigate this. Response-adaptive designs may further reduce harm, especially for binary outcomes when early efficacy signals arise without prior toxicity concerns. | Emergency situations may impair parental autonomy due to stress and time pressure. Antenatal counselling can help, while Department of Health and Human Services (DHHS) and FDA frameworks allow consent waivers in high-risk research. | Equitable participation is key but must not be used to exploit vulnerable populations. Recruitment methods must avoid overrepresentation of certain socioeconomic groups. |
| Paper 3: Anand et al, Analgesia and anesthesia for neonates: study design and ethical issues (29) | | | | | | |
| Literature review exploring the ethical challenges of neonatal analgesia trials, including study design. | | PCTs can advance neonatal care but must ensure benefits outweigh pain risks. Placebo groups require close monitoring and predefined withdrawal rules. PCT design strengthens validity. | | Placebo is unethical in pain trials when effective treatments exist as untreated pain harms neurodevelopment. As in asthma or hypertension, withholding care poses risks. Add-on designs with pain protocols for placebo groups are vital. | Neonates cannot consent; parents have to make decisions under stress. Thorough explanation is important. Variability of review board interpretation of national guidance is a concern. | Neonates need access to evidence-based care. Trials should target key research gaps, ensure diverse recruitment, and limit reimbursement to essential costs to avoid financial coercion. |
| Paper 4: Axelin et al, Ethics in neonatal pain research (30) | | | | | | |
| Literature review on ethics of neonatal analgesia trials, including consent, ethical approval, placebo and journal reporting. | | While minor harm is a risk, neonatal pain research has brought important benefits to infants. | | In 75% of studies, infants experienced pain when placebo or insufficient pain relief was used. Declaration of Helsinki must not be compromised by national or regulatory differences. | Informed consent obtained in 94% of studies reviewed. Some countries require consent from both parents (practice varies). Authors are doubtful of whether parental consent is truly informed in a stressful situation. | Paediatric research was once seen as harmful, but denying children research benefits can be viewed as more damaging. Equal rights to safe, evidence-based care make research essential. |
| Paper 5: Baer et al, Ethical challenges in neonatal research: summary report of the ethics group of the newborn drug development initiative (14) | | | | | | |
| Commentary from Newborn Drug Development Initiative workshop summarising ethics of developing medicines in neonates. | | Neonatal research is essential to reduce off-label drug use (e.g. phenobarbital for seizures) and replace dependence on unproven treatments. “Standard” therapy often lacks efficacy and safety data. | | PCTs (e.g. involving neonatal pain, cardiovascular instability) risk avoidable harm. Placebo should be used if no proven therapy exists or risk is minimal. Rescue protocols are essential to minimise harm. | Parental consent during emergencies is compromised and less likely to be fully informed. Ongoing communication with parents during trial important. | Variability in neonatal intensive care practices (e.g. methylxanthine use) undermines research equity. Care should be standardised by IRBs across sites via collaborative networks. Clinicians and parents must help design trials. |
| Paper 6: Bellieni et al, Recommendations for an ethical treatment of newborns involved in clinical trials (36) | | | | | | |
| Guidance for judging neonatal research ethics, including placebo use and blood sampling. | | Paediatric research is important to help all children, who have interests which are different from adults. | | 15/18 trials included in study period (2007-9) used placebo or ineffective analgesia in controls. Ethical research must minimise pain, use validated analgesia in comparisons, limit invasive procedures and avoid publishing unethical studies. | Fully informed consent essential. | Newborns have specific needs which must be accounted for when planning clinical research in this cohort. |
| Paper 7: Bellieni et al, Should an IRB approve a placebo-controlled randomized trial of analgesia for procedural pain in neonates? (37) | | | | | | |
| Ethics of neonatal pain trial proposal submitted to IRB discussed by pain and palliative care specialists. | | Placebo use in clinical trials may be deemed acceptable if tackling a critical “unreolved issue”. | | Placebo denies neonates of known effective analgesia, causing unnecessary suffering. New treatments should be compared against current gold-standards with proven effectiveness (e.g sucrose/breast milk). | Adult consent based on what they could tolerate is not sufficient to justify withholding analgesia. Ethical standards should be especially stringent in this age group. | Neonatal care standards vary (e.g. sucrose use from 5-100%). Since sucrose is not consistently used as standard care, a PCT would not be denying established treatment. High-quality practices should be more widely adopted. |
| Paper 8: Bellieni et al, Analgesia, nil or placebo to babies, in trials that test new analgesic treatments for procedural pain (51) | | | | | | |
| Assess how often neonates in control groups of analgesia trials were denied effective pain relief during painful procedures; encourage ethical reform. | | Not discussed. | | 64% (29/45 RCTs) of control groups received placebo/no analgesia. Minor procedures can cause physiological stress and long-term harm. Avoidable pain in trials breaches ethical norms (e.g. Declaration of Helsinki). Validated analgesics (e.g. sucrose) are available. | Infants cannot consent; parental consent may be uninformed or influenced by researchers. The authors argue parents should only consent to the least harmful option. | Neonates, a highly vulnerable group, were repeatedly exposed to pain without analgesia despite guidelines. The authors urge ethics boards and journals to reject these unjustified study designs. |
| Paper 9: Desselas et al, Drug versus placebo randomized controlled trials in neonates: A review of ClinicalTrials. gov registry (31) | | | | | | |
| Review of ClinicalTrials.gov registry (1999–2015); overview of placebo use in neonatal PCTs. | | Placebo justified only when no standard treatment exists. Premature infants differ physiologically from adults’ age-specific research needed to ensure safe and effective care. | | In serious conditions, placebo may be a “loss of chance,” especially where no benefit is expected. Of 15 neonatal placebo RCTs reviewed (1999-2015), 9 involved painful procedures with no rescue therapy. | Not discussed. | Around 90% of neonatal drugs are used off-label with inconsistent dosing. Industry-sponsored trials in neonates are rare. All neonatal PCTs should be registered to ensure transparency and equitable representation. |
| Paper 10: Theubo et al, No pain, neurodevelopmental gain: Time to avoid painful placebo injections in neonatal research (52) | | | | | | |
| Consider the use of placebo-controlled RCTs in neonatal studies involving painful procedures. | | High-quality care for both preterm and term neonates with birth complications has significantly improved through robust RCTs and meta-analyses. | | Use of saline placebo injections in preterm trials should be re-evaluated. Pain can negatively affect neurodevelopment, and using intramuscular or intravenous placebos is no longer ethically justifiable. | Neonates cannot consent, so participation relies on caregiver consent. Parents often agree knowing the research may benefit future infants and that their child will be protected under the principle of minimal risk. | Not discussed. |

**Table S2:** A summary table of the included paediatric studies and the key themes raised according to the 4 principles of clinical ethics.

| **Aim / Summary** | **Beneficence** | **Maleficence** | **Autonomy** | **Justice** |
| --- | --- | --- | --- | --- |
| Paper 1: Anderson et al, The placebo (I shall please)–is it so pleasing in children? (19) | | | | |
| Outline ethical use of placebos in paediatric trials, with recommendations and consideration of children's vulnerability. | Drug disasters like thalidomide show the need for rigorous paediatric trials. Priorities include child wellbeing, low invasiveness (e.g. minimal blood tests), and clear benefit. The Declaration of Helsinki allows placebo use only if no standard treatment exists and equipoise is present. | Children’s vulnerability redefines minimal risk. Ages 2–6 often find venepuncture distressing; neonates risk anaemia. Pain is less acceptable than in adults. Modelling can reduce invasive tests. Some therapies pose long-term risks (e.g. cancer, developmental harm). Crossover/adaptive/add-on designs preferred to withholding established treatment. | Children under 9 often don’t understand trial aims or risks. Assent is important but may be influenced by pressure, and withdrawal rights may be unclear. Ethics committees guide decisions, though approaches vary internationally. | To avoid therapeutic orphanhood, children must be included in robust trials. FDA incentives support this but need ethical balance. Cultural differences influence trial design; global cooperation can help. Placebos aren’t always needed to see benefit, as seen in oncology. |
| Paper 2: Armenteros et al, Do we need placebos to evaluate new drugs in children with schizophrenia? (26) | | | | |
| Literature review of paediatric schizophrenia PCTs and key guidelines (e.g. Helsinki, DHHS, AAP) to assess ethical and scientific grounds for placebo use. | Placebo can isolate drug effects, especially with high response rates in schizophrenia (up to 22%). Adult data isn’t applicable due to developmental differences. Only two paediatric PCTs exist. Active drugs may cause serious side effects like agranulocytosis or tardive dyskinesia, supporting use of placebo to compare overall benefit. | Risks include symptom worsening during placebo, and possible neurodevelopmental harm from untreated schizophrenia. Trials must monitor closely, shorten placebo use (despite limited long-term data), and focus on acutely unwell patients to minimise harm. | Cognitive and developmental limits in paediatric schizophrenia affect autonomy. Ideally, obtain parental consent and child assent, but guardianship may be needed if illness impairs capacity. | Children with schizophrenia are often excluded from research, limiting access to tailored treatments. PCTs generate age-specific safety and efficacy data. Post-trial access to effective therapies ensures fair benefit. DHHS allows such trials if they produce generalisable knowledge on serious conditions. |
| Paper 3: Atuire et al, COVID-19 vaccine trials with children: ethics pointers (32) | | | | |
| Ethical guidance on phase III COVID-19 vaccine trials involving children in low/middle-income countries (focus on consent, placebo use, and trial design). | Vaccines reduce risk of disease in children and any associated long-term complications. Vaccination also facilitates safer social interactions which is crucial for development and school attendance. PCTs are valuable because children are physiologically and developmentally different from adults. | Placebo could delay protective or life-saving interventions in global emergencies. Of particular concern is if a vaccine already exists in the target age group or population. | Promotes use of assent in children, which must be carefully designed by ethical committees to be age-adjusted and culturally sensitive. | Key is to ensure equitable access and inclusion of children of all ages and demographics across the globe. This is important to address the global disparity in accessing vaccines. |
| Paper 4: Auby, Ethical, clinical and practical considerations in designing clinical trials in pediatric psychopharmacology (38) | | | | |
| To discuss ethical, clinical, and practical considerations in designing paediatric psychopharmacology trials. | Paediatric mental health needs better treatments; RCTs are better at identifying causal patterns. Research provides safe access to new therapies and may reduce off-label use. Placebo is justified when clinical equipoise exists. SSRI PCTs show modest benefit and safety concerns (e.g. suicidality), supporting the need for robust PCTs. | Poorly designed PCTs carry the highest risk. Inconsistent definitions of ‘minimal risk’ may hinder vital research or harm children unnecessarily. Placebo may delay treatment in severe cases, and long-term safety remains hard to assess. | Informed consent is essential. The American Academy of Pediatrics suggests assent may be possible from an intellectual age of 7. Other sources suggest ages 8–14, with adult-level understanding typically by 14. | Children with mental disorders should benefit from research and innovation. Global disparities must be addressed, as echoed by the World Health Organization’s 2007 ‘Make Medicines Child Size’ campaign. |
| Paper 5: Berde et al, Pediatric analgesic clinical trial designs, measures, and extrapolation: report of an FDA scientific workshop (53) | | | | |
| Expert consensus at an FDA scientific workshop, discussing the scientific, ethical and practical considerations for conducting analgesic trials in children. | Efficacy of a new analgesic cannot be reliably established without a placebo-controlled trial. | Placebo arms without pain relief are unethical. Rescue analgesia can act as an endpoint, preserving trial rigour while minimising suffering, though this is hard to sustain for prolonged pain. Pharmacokinetic and safety studies are vital, especially for drugs with unclear mechanisms. | Adults can consent to placebo contolled analgesia trials but children should not. | Conducting trials in every age group isn’t always feasible. Adult data may be extrapolated when the drug’s mechanism is well understood and evidence-based (e.g. m-opioids, NSAIDs and acetaminophen in children over 2 years old). |
| Paper 6: Braga et al, Placebo-controlled trials in pediatric urology: a cautionary view from an ethical perspective (2) | | | | |
| Narrative review of ethical issues in paediatric urology placebo-controlled trials, including specific indications for their use. | PCTs in paediatric urology are justified when no standard treatment exists, care is ineffective, new evidence challenges practice, or treatment is inaccessible. Some “proven” therapies (e.g. high oxygen in preterms) have been shown to carry risks. PCTs clarify benefit efficiently but require true equipoise; ethics comes before trial cost/simplicity. | Non-therapeutic risks (e.g. injection or surgical discomfort) must be minimised. Surgical placebos raise ethical concerns due to higher risk. Trials should involve no more than minimal risk, as judged by ethics boards. Burdens like follow-up are more acceptable than harm. Placebos are unethical if life-saving treatment exists. | Researchers must clarify that trial participation may not benefit the child directly, to avoid therapeutic misconception. PCTs can be ethical if harm is unlikely, ethics approval is granted, and all options are explained. Assent should be sought from ages 7–15 as per international guidance. | Fewer than 1% of paediatric urology studies are RCTs. Ethics boards must balance risk with children’s right to benefit from research. Without this, children risk remaining “therapeutic orphans” excluded from medical advances. |
| Paper 7: Coffey et al, Ethical assessment of clinical asthma trials including children subjects (35) | | | | |
| Systematic review including US based asthma trials assessing whether children with asthma were harmed by participation in placebo-controlled trials. | None noted. | In 45 asthma PCTs, 19% withdrew, more in placebo arms (15% vs 6.5%) due to exacerbations. Only one study analysed children separately. Withholding steroids worsened symptoms, raising ethical concerns, especially where clinical uncertainty was lacking. | 68/70 trials documented informed consent (role of child in consent not discussed). | The 2002 Declaration of Helsinki permits fair placebo use when scientifically justified for minor conditions, as long as it poses no serious or irreversible harm. |
| Paper 8: Danese et al, Are we ready to abandon placebo in randomised clinical trials for inflammatory bowel disease? Pros and cons (33) | | | | |
| Panel summary from 2015 European Crohn’s and Colitis Organisation Congress covering ethics of placebo, recruitment challenges, and alternative trial designs. | Placebo-controlled designs aid early detection of effective treatments (minimising exposure to ineffective treatments), help isolate background drug side effects, and can reduce trial size and duration. | Delayed treatment in placebo arms (e.g. higher colectomy rates in ulcerative colitis) raises ethical concerns, especially for children where growth may be affected. Rescue plans and biomarker-based designs can help reduce risk. | Children may struggle to understand the risks of chronic illness. Including those aged 14 and above in adult IBD trials may be appropriate, as defined by the US National Institutes of Health. | Recruitment issues may cause selection bias, with sicker patients more likely to enrol onto a placebo controlled trial. Adult data and biomarkers may support smaller, fairer paediatric trials. |
| Paper 9: Derivan et al, The ethical use of placebo in clinical trials involving children (6) | | | | |
| Panel review of ethical and regulatory guidance on paediatric placebo use, with recommendations proposed. | Paediatric research is vital where no proven treatment exists. Common therapies may lack solid evidence in children. Placebo is useful in conditions with strong or age-dependent placebo effects (e.g. ADHD, depression). Run-in phases may help identify non-responders in minor conditions. | Placebo use in severe conditions risks harm and should be time-limited. Rescue therapy is essential. While avoiding harm is key in paediatric trials, it can be difficult to define without first understanding what causes harm. | Informed consent essential. | Belmont report emphasises respect and justice, and that the “burden” of research should be distributed equally across society. |
| Paper 10: Di Pietro et al, Placebo-controlled trials in pediatrics and the child’s best interest (25) | | | | |
| To evaluate ethical challenges of placebo use in paediatric trials and promote child-centred study design. Review regulations (e.g. Paediatric Regulation, Helsinki, AAP) and examples. | Clinical equipoise is key. Placebo is acceptable only when no proven treatment exists. High placebo response in child psychiatry complicates drug assessment. Off-label use risks poor adherence. PCTs need fewer patients, cost less, and may speed access to effective treatments. | Replacing off-label therapies with placebo in serious illness may cause harm, especially with treatment interruption. Risks are higher in oncology trials. To reduce harm: exclude high-risk patients, shorten placebo use, monitor closely, and stop for adverse events. Comparing to off-label treatments may be safer. | Therapeutic misconception is common, consent forms often lack full placebo details. Children over 9 (especially 14-18) can often join decisions. If not, choices should reflect their best interest. Physicians must avoid bias. AAP highlights that quality-of-life judgments are complex and should include the child’s view when possible. | Children are at higher iatrogenic risk from off-label drugs and need access to well-tested treatments. Better methods are needed to compare new drugs with effective off-label options, not just placebo. |
| Paper 11: Faria et al, Parental attitudes about placebo use in children (54) | | | | |
| To obtain parental opinion about the use of placebo in paediatric RCTs and clinical practice. | A 2014 survey of 1000 parents; 91.5% supported placebo use in paediatric trials, 76.6% would enrol their child knowing that placebo was a possibility. Acceptance was highest for psychological therapies and influenced by transparency, safety, and doctor’s advice. 72.5% preferred PCTs over ACTs if side effects were a concern. Stronger placebo responses in children support ethical use with consent. | 5.7% felt they placebo is never acceptable. Parents do not agree with any placebo used deceptively. | Placebo role should be fully explained so parents can make better informed decisions. | Over half of drugs used in paediatrics utilise data from adult population which may affect safety/efficacy data when extrapolated to children. |
| Paper 12: Flynn, Ethics of placebo use in pediatric clinical trials: the case of antihypertensive drug studies (5) | | | | |
| To explore ethical issues in paediatric antihypertensive placebo trials and develop guidance for trial design. | Placebo may be ethical in mild, asymptomatic hypertension without organ damage or secondary cause, for short durations (4–8 weeks), when no standard treatment exists. Requires close monitoring, clear stop rules, and ethics panel oversight. | Withholding treatment may harm children, especially in severe hypertension or with organ damage. Up to 25% have left ventricular hypertrophy at diagnosis. Placebo groups had double the withdrawal rate. Most paediatric hypertension is secondary; adult data supports treatment, so placebo use may be risky. | The IRB have responsibility to protect children as parent’s decision may not always be in child’s best interest. | Important to move away from children as “therapeutic orphans” by developing ethically sound and robust RCTs in children, which may require placebo. |
| Paper 13: Fost, Ethical issues in research and innovative therapy in children with mood disorders (17) | | | | |
| To explore ethics of PCTs and new therapies in paediatric mood disorders, using cases and frameworks. | Placebo effect more common in context of mood disorders. No toxic side effects of placebo compared to other therapies. Promoted when clinical equipoise and no other treatment exists. | Children could be harmed from their illness in the placebo group. Suggest cross-over design to combat this. | Children who are mature enough to consent may still be vulnerable to exploitation, under pressure that they ought to participate to help the community. | Justice involves including children in medical progress. Not including children in RCTs delays knowledge about how to treat paediatric medical conditions, which is not in the best interests of children as a population. |
| Paper 14: Hawkins, Justice and placebo controls. Social theory and practice (55) | | | | |
| Review of placebo ethics through two examples: surfactant vs. placebo in preterm infants, and azidothymidine vs. placebo to prevent mother-infant HIV transmission. | Compared researchers with ‘Good Samaritans’, who have a duty to act in the best interests of trial participants. | In the surfactant trial, placebo was needed for valid results, but critics argued it served manufacturers more than patients, as surfactants were already known to be effective. The article compares withholding treatment to bystanders watching a child drown. | Not discussed. | Not discussed. |
| Paper 15: Jacobson et al, Testing vaccines in pediatric research subjects (34) | | | | |
| Literature review and discussion identifying ethical concerns and practical alternatives in paediatric vaccine trials. | The AAP outlines five scenarios in which placebo use in paediatric research may be ethically justified: no standard treatment; existing treatments lack efficacy; current interventions pose safety concerns; combining treatments could be harmful; or disease severity differs significantly between individuals. | While methodologically useful, placebo use in children raises ethical concerns. To reduce perceived risk, researchers often use active or non-therapeutic comparators to maintain blinding, though these still carry ethical tension. | Recent studies have shown that using alternative agents instead of placebos can preserve study integrity while improving acceptability to parents. Assent important where appropriate. | Paying child participants in research involves ethical and regulatory complexities. Though placebo use is allowed, researchers often use alternative comparators to support enrolment. |
| Paper 16: Solodiuk et al, Balancing ethics and science in pediatric pain intervention trials (56) | | | | |
| Summarise challenges in infant pain trials (including placebo), using study comparing sucrose, radiant heat, and pacifier for vaccination pain. | Efficacy comparisons between treatments can be misleading; being similar to an effective treatment doesn’t guarantee superiority over placebo. | The article concludes with a reminder: research should ultimately serve humanity, not just scientific progress. | Since children cannot provide informed consent, placebo use is generally avoided when successful treatments are already available. | Excessive caution in enrolling children in trials has slowed progress in paediatric care. |
| Paper 17: Kelly et al, Important issues in the justification of a control treatment in paediatric drug trials (57) | | | | |
| Explores challenges in choosing comparators in paediatric trials and proposes criteria for placebo or active control use, based on 57 paediatric plans submitted to the EMA. | 28% of plans had comparator issues, prompting a justification tool. Placebo may be ethical when added to standard care (not used alone) if effective treatments exist. | International guidance allows placebo in children only if withholding treatment poses no serious risk (e.g. death or lasting harm). Developmental impact must be considered. Placebo is unethical if proven therapies improve survival or prevent serious harm. | Engaging patients or caregivers can enhance relevance and acceptability. | Not discussed. |
| Paper 18: Knellwolf et al, Framework conditions facilitating paediatric clinical research (7) | | | | |
| Discuss framework conditions facilitating paediatric research; comparisons of European Union regulatory bodies with US FDA. | Ethical use of placebo may be justified in situations such as: lack of effective standard treatments; failure of existing therapies; concerns over effectiveness or safety of current options; use to measure new treatment side effects; or when the disease is unpredictable and current care is unproven. | Ethical concerns about clinicians’ limited research training and parents’ misunderstandings. Add-on and withdrawal trials suggested as an alternative to placebo-only trials. | The ability to understand research may begin around age 9. Criminal responsibility begins as early as age 7 (Switzerland), 11 (UK), and 14 (Italy), yet assent may still be overlooked in research. The Oviedo Convention urges increasing weight to minors’ views with age. | 50-80% of medicines used in children remain unlicensed or untested for paediatric populations, despite children comprising about 25% of Europe’s population. Paediatric research should be viewed as a vital opportunity to improve child health. |
| Paper 19: Lim et al, Is there a place for placebo in management of psychogenic movement disorders? (58) | | | | |
| Explores ethical issues of placebo use, focusing on a 17-year-old with psychogenic blepharospasm treated with a disguised inert substance. | Placebo effects tend to be stronger in psychological conditions. | The case raises questions about placebo in clinical care and trials. The Declaration of Helsinki favours comparing new treatments to standard care over placebo. Risky procedures like sham surgery are unethical if they offer no potential benefit to participants. | Emphasises importance of autonomy | Not discussed. |
| Paper 20: Linde et al, Ethical aspects of placebo in migraine research (21) | | | | |
| Discusses migraine trial ethics in light of the Declaration of Helsinki, covering trial designs, placebo challenges and use of rescue medications. | Placebo trials help confirm efficacy when existing treatments are weak. Non-inferiority (active control) designs may be less reliable than superiority trials, even with larger samples. Expectation of active treatment can inflate placebo response. | Ethical standards must override national laws. PCTs prioritise statistical significance over care, but ACTs may need larger samples and pose greater risk. A (50:50) placebo design gives strong data but delays treatment; active controls ensure access. Migraine trials often use early escape with rescue meds after 2–4 hours. | Consent must be meaningful to respect personhood. The revised Declaration of Helsinki allows including minors or incapacitated adults only when essential, unlike other guidelines, which presume inclusion unless exclusion is justified. | The Declaration of Helsinki restricts placebo use when effective treatments exist, but this is not fully echoed by regulators like the FDA. |
| Paper 21: Lynch, Give Them What They Want-The Permissibility of Pediatric Placebo-Controlled Trials under the Best Pharmaceuticals for Children Act (15) | | | | |
| Explores ethical and legal issues of placebo use in paediatric trials, with reference to Best Pharmaceuticals for Children and Pediatric Research Equity Act implemented by the FDA. | PCTs matter because children differ physiologically from adults. Off-label use (e.g. SSRIs) can be unsafe. With up to 65% of paediatric prescriptions lacking safety/efficacy data, trials are essential. Placebo is appropriate when equipoise exists and no proven treatment is available. | Withholding treatment in PCTs may harm children, particularly in conditions like depression where equipoise is unclear. Psychological risks are higher due to vulnerability and limited understanding. Risk must be minimised and benefit maximised. | Children cannot give full informed consent and rely on parental consent and their own assent. They may be vulnerable to coercion if parents have personal motives. Independent review boards play a key role in protecting autonomy. | Limited financial incentives hinder paediatric trials, raising off-label use risks. Vulnerable groups, like institutionalised children, may be unfairly burdened. Fair inclusion across age groups is needed, and active-control trials may be a more just option. |
| Paper 22: March et al, AACAP 2002 research forum: placebo and alternatives to placebo in randomized controlled trials in pediatric psychopharmacology (24) | | | | |
| To explore placebo use in paediatric psychopharmacology trials. Workgroups examined challenges and solutions across ethics, trial design, consumer input, regulatory views, and psychosocial options. | Placebo use may be fair when risk is low, no proven treatment exists, or better efficacy data is needed. It is justified by poor adherence, unclear long-term safety, or unreliable usual care, especially in psychiatric trials where evidence is weak. Acceptability improves with flexible design and stakeholder input. | Placebo should be avoided when withholding treatment risks lasting harm. “Nocebo effect” can lead to adverse consequences. Children may also suffer if treatment delayed due to placebo. | Research requires stricter consent than clinical care. The Belmont Report stresses that informed consent must go beyond risks, it should clarify the difference from regular care and highlight alternatives. Long consent forms may still fail to achieve true understanding. | Placebo may be considered more ethically acceptable when commonly used treatments are inaccessible due to cost or system barriers. |
| Paper 23: McGuirk et al, Use of invasive placebos in research on local anaesthetic interventions (59) | | | | |
| To assess whether placebo controls in local anaesthetic trials pose harm to participants and to introduce a new tool to categorise risk severity. Developed the ‘SHAM’ (Serious Harm and Morbidity) scale. | Blinding may improve trial quality but often failed to justify the risks, especially in children. Use of placebo increases trial validity. | Review included 59 RCTs (10 paediatric), using the SHAM scale (0 = no risk to 4 = major risk). 53% scored SHAM ≥3, including six studies involving 183 children who faced moderate or major risks. Several placebo interventions carried serious complications such as neuropraxia or blindness, raising concerns about compliance with the Declaration of Helsinki. | Informed consent was obtained, but asking parents to consent to invasive, non-beneficial procedures raises ethical concerns. | Ethical protections were inconsistently applied; vulnerable groups like children were not uniformly safeguarded. |
| Paper 24: Momper et al, Ethical considerations for pediatric placebo-controlled trials: FDA outcomes and perspectives (3) | | | | |
| Explores FDA oversight of paediatric PCTs under 21 Code of Federal Regulations (Part 50, Subpart D), which limits children’s exposure to non-beneficial research risks. | EU guidance supports placebo only when genuine uncertainty exists, as the most rigorous method to test therapies. | Review of 96 paediatric PCTs found that most used risk-reduction strategies, such as short placebo exposure, rescue medication, or add-on designs. Ethical acceptability depended on trial duration, delivery method, and the presence of alternatives. When risks couldn’t be minimised, justification relied on minimal harm or clinical need. | Not discussed. | Not discussed. |
| Paper 25: Morales-Olivas et al, Clinical trials in children (16) | | | | |
| Literature review exploring paediatric drug responses, ethical concerns (e.g. consent, placebo use), trial design, and the risks and benefits of paediatric trial participation. | Trial participation may improve care through closer monitoring. Placebo is inappropriate when effective treatments exist, but in paediatrics, unclear standards complicate ethics. Enrolling well or at-risk children in non-therapeutic studies may still be fair. | Paediatric dosing is complex and often guided by growth-based drug models. Trials raise specific concerns; pain, anxiety, caregiver disruption, and growth impact. Higher-risk studies may be fair if direct benefit is likely. | Children are legally defined as under 16 (US) or 18 (EU) for research consent. Some believe off-trial treatments are safer, though trials offer better monitoring. Parents often grasp risks more than benefits; improved understanding builds trust in research. | The phrase “therapeutic orphans” was coined in 1968 to reflect the scarcity of tested, child-appropriate medications. Limited financial incentives and small paediatric markets often deter pharmaceutical companies from investing in trials for children. |
| Paper 26: Perez et al, Parental perceptions of research after completion of placebo-controlled trials in pediatric gastroenterology (60) | | | | |
| Compares views on research between parents of children who completed placebo-controlled paediatric gastroenterology trials and those who withdrew or were excluded. | 78 parents responded to a 26-item questionnaire 6 months after trial. Most parents, regardless of trial completion, held positive views of research. 49% were comfortable with potential placebo use. Parents believed research was important (99%), felt their child’s interests were prioritised, and reported satisfaction even without direct benefit. | 22% of parents expressed discomfort with the idea of their child receiving placebo. | Most parents (92%) felt free to withdraw, with few feeling pressured to consent (10%). Satisfaction was linked to clinician rapport and clarity of the consent process. Consent processes were viewed as clear and sufficient time given to make a decision. | Not discussed. |
| Paper 27: Pinxten et al, Frontline ethical issues in pediatric clinical research: ethical and regulatory aspects of seven current bottlenecks in pediatric clinical research (61) | | | | |
| Discussion of literature and regulations. Highlights seven challenges in paediatric research ethics, one of which is placebo regulation. | Placebo use is the gold-standard but must be regulated. One “golden rule” does not apply to all cases. PCTs may offer benefits such as fewer participants, stronger data and structured care. While placebo groups may face greater risk of no benefit, overall harm may be reduced. | Randomisation can be hard for families to grasp. Withdrawing treatment may reduce care quality and breach equipoise, which requires all study arms to offer similar potential benefit. | Presenting research within the “therapeutic context” of each patient can help clinicians, children, and their families make well-informed decisions about trial participation. | In paediatrics, research and therapy often overlap, as both trials and off-label use involve experimental treatments. Trials may not increase risk and can offer benefits like close monitoring and ethical safeguards. Banning placebo use could hinder evidence-based care and worsen the “therapeutic orphan” problem. |
| Paper 28: Robinson, Ethical issues in pediatric research (62) | | | | |
| Outlines how an IRB should assess a study protocol, using a hypothetical example involving premature infants receiving IM injections of the study drug. | Paediatric research is allowed if risks are minimal, slightly above minimal with valuable knowledge, or justified by benefit. | IRBs must justify withholding treatment from the placebo group. The dilemma is that the trial’s purpose is to determine this balance. For non-cognitive infants, a placebo injection may be unnecessary; using a bandage can blind observers without causing pain. | Informed consent is central to research ethics, but children cannot legally provide it. Instead, parental permission and the child’s assent are required. The Declaration of Helsinki supports “proxy consent,” but this remains ethically and practically complex. | Excluding children from medical research denies them of the same opportunities that adults get to be safety treated. |
| Paper 29: Rose et al, Ethical issues in pediatric regulatory studies involving placebo treatment (63) | | | | |
| To review the regulatory barriers to antiepileptic drugs in children. Critique of the regulatory environment for paediatric drugs. | Since the 1960s, antiepileptic drugs have required placebo controlled trials for approval worldwide. This was to avoid recurrence of harm in the paediatric population caused by off-label drugs. | The Declaration of Helsinki permits research only to advance medical understanding. The authors argue that re-testing known drug efficacy in children often lacks ethical justification. Financial incentives have led to an ongoing cycle of paediatric trials, fostering what the authors describe as an “academic industry” reliant on repetitive studies. | Regulatory definitions may label 12–17-year-olds as paediatric, but they are often physiologically mature. | The term “therapeutic orphans” emerged in response to FDA warnings that excluded children from trials, driven by safety concerns and legal protection. This exclusion spurred growth in child-specific research, aligned with the view that children aren’t small adults and the rise of children’s rights. |
| Paper 30: Rose et al, Questionable industry-sponsored postneonatal pediatric studies in Slovenia (64) | | | | |
| Review of paediatric trials in Slovenia found some ethically questionable studies, often driven by regulatory incentives rather than clinical need or clear benefit. | In some conditions, like hypertension, children may react differently to medications due to factors like greater vessel elasticity. However, for most diseases, organ maturity after birth permits treatment using similar approaches to adults. | Trials lacking meaningful clinical questions raise ethical issues. In paediatrics, regulatory demands have increased industry-led studies, often influenced by academic and commercial incentives. | FDA and European regulations define children as under 17 and 18 years, respectively. They argue this overlooks adolescents’ capacity to make autonomous decisions, particularly in cases like healthy 16-year-olds who may be developmentally similar to adults. | Grouping all under-18s together in regulatory definitions fails to account for developmental differences, resulting in unnecessary restrictions on adolescents’ access to appropriate care or inclusion in meaningful research. |
| Paper 31: Sammons et al, British and Canadian views on the ethics of paediatric clinical trials (39) | | | | |
| Survey of 100 UK and Canadian researchers (66% doctors) explored ethical views in paediatric research, (including placebo use), with questions and trial scenarios. | Not discussed in the placebo scenarios. | Placebo use raised concern, especially with analgesics. Over two-thirds objected to testing drugs in healthy children. Nearly half supported enrolment in a placebo-controlled antibiotic trial for otitis media, though many preferred active or standard treatment comparisons. | There was consensus among respondents that children as young as 10 years (interquartile range, IQR, 7–12) might be capable of assent, while full consent was generally considered appropriate from around 14 years (IQR 12–16). | Not discussed in the placebo scenarios. |
| Paper 32: Sammons et al, Ethical issues of clinical trials in children (20) | | | | |
| Review discussing challenges and necessities of research in children. | Ethical paediatric trials are vital to improve child health, especially where evidence is limited. Trials should minimise risk and target unmet needs, with benefits including better safety, dosing accuracy, and child-friendly formulations. | Children are not small adults; their conditions and drug responses differ. Research must balance benefit and risk, from low-risk checks to high-risk procedures. Population pharmacokinetics reduces sampling needs. EU rules limit blood draws (2.4 ml/kg over 4 weeks) and generally ban research on healthy children, except for vaccines or taste studies. | The AAP recommends seeking assent from age 7; elsewhere in Europe, the range is 7–16. Studies suggest many children understand risks and benefits by age 9. In emergencies, research may begin without consent, but both parental consent and child assent should be obtained as soon as feasible. | EU rules prohibit financial incentives for children in trials but allow reimbursement. Conducting clinical research in children is vital to improving paediatric health outcomes. As adverse drug reactions can vary by age, it is necessary to evaluate medicines across the full paediatric age spectrum. |
| Paper 33: Sandler, Placebo effects in developmental disabilities: implications for research and practice (22) | | | | |
| To explore the implications of placebo effects in children with developmental disabilities. | Placebo justified when scientifically necessary, low risk and fully consented. Placebo effects are common in children with developmental disabilities; around 30% respond in ADHD trials. In autism, natural behavioural changes may be misattributed to treatment. While placebos affect perception more than the disease, they can still offer clinical benefit. Run-in phases help identify responders. | Placebos are unethical if they withhold essential treatment. | Deceptive use is rejected and does not respect autonomy; open-label placebo use is supported. | Banning placebo entirely could limit evidence-based care. |
| Paper 34: Scahill et al, The science and ethics of placebo in pediatric psychopharmacology (65) | | | | |
| Discuss design of drug trials in psychology. Reviewed trial designs and ethical frameworks. | Placebo use in paediatric psychopharmacology may be justified for conditions like autism or Tourette syndrome, where treatments are limited or have significant side effects. Ethical justification relies on equipoise, minimal risk, and potential benefit. | PCTs risk withholding treatment, though open-label follow-up may help. Blinding and defining relapse are difficult. Active comparator and discontinuation trials have limits. Pilot studies help with design but may yield misleading results. Superiority to placebo isn’t required. | Not discussed. | Applying strong ethical and scientific standards is essential to ensure clinical trials are reliable and avoid unclear results that fail to inform practice. |
| Paper 35: Turner et al, Designing clinical trials in paediatric inflammatory bowel diseases: a PIBDnet commentary (44) | | | | |
| Formulate trial design in paediatric IBD to reduce time lag between drug trials in adults and children. A global expert group reached consensus on 18 statements to guide ethical, feasible trial design in paediatric IBD. | Placebo is only justifiable for new drug classes when no approved paediatric treatments remain, and true equipoise exists in and outside the trial. Dosing and safety data can’t be assumed from adults. | Children with IBD shouldn’t receive placebo if the drug has already shown superiority in adults or children. PCTs should follow open-label induction to full remission, with early escape plans for relapse. Adolescents may join adult placebo trials only if standard treatments have failed, and subgroup size must allow meaningful analysis. | Striking the right balance between study quality and practicality is harder in paediatrics, as parents can't consent solely for altruism or financial gain. | Delays in paediatric drug approval prolong off-label use, and dosing extrapolated from adults often leads to underdosing in children. |
| Paper 36: Turner et al, Use of placebo in pediatric inflammatory bowel diseases: a position paper from ESPGHAN, ECCO, PIBDnet, and the Canadian Children IBD Network (45) | | | | |
| Define when placebo is acceptable in paediatric IBD trials. Position paper by a group of experts. | Placebo may induce clinical remission in up to 20% of paediatric IBD cases, but emphasises that this benefit is minimal or absent when using objective outcomes like mucosal healing. Placebo should only be considered when there is genuine uncertainty about whether a treatment is effective. | Assigning children to inferior treatments, particularly when more effective options are already accepted in paediatric care through adult data extrapolation, raises serious ethical concerns. | Since children depend on adults to make decisions in their best interest, parents are not permitted to consent to a placebo-controlled trial solely to benefit future research. Unlike autonomous adults, children cannot ethically be enrolled for altruistic reasons alone. | Paediatric IBD trials must be timely and robust to reduce delays in drug availability for children. Studies should compare against active treatments and focus on dosing and metabolism, especially those aged 2–11. Given the greater disease burden in children, trials should not expose them to unnecessary risk by denying effective care. |
| Paper 37: Vitiello, Ethical considerations in psychopharmacological research involving children and adolescents (23) | | | | |
| Review and discussion of ethical considerations of psychopharmacological research in children and adolescents. | In adolescent depression, placebo response rates of (40–50%) suggest potential therapeutic benefit. Placebo use may be justified when effective treatments or long-term safety data are lacking. Since psychotropic effects in children differ from adults, paediatric trials are essential. | Any potential risks must be weighed against the consequences of not treating psychiatric conditions during critical developmental periods. | For studies that do not offer direct benefit or involve more than minimal risk, consent is typically required from both parents. Children around the age of 7 or older are generally able to assent, supported by age-appropriate forms alongside parental consent. | Overall risk–benefit balance must clearly favour the child and be at least equal to current alternatives. |
| Paper 38: Waubant et al, Clinical trials of disease-modifying agents in pediatric MS: opportunities, challenges, and recommendations from the IPMSSG (42) | | | | |
| Experts from the International Pediatric multiple sclerosis (MS) study group proposed trial strategies to generate evidence for paediatric MS therapies. | If supported by biological and pharmacological data, paediatric MS approvals may rely on open-label pharmacokinetic/pharmacodynamic and safety studies. Where more evidence is needed, brief trials using MRI endpoints (rather than clinical relapses) may suffice. | In paediatric MS, placebo is unethical when adult treatments are proven. Add-on trials offer an ethical way to maintain access to effective care during research. Families did not agree with placebo injections. | Children had a desire to contribute to science however parents demonstrated more caution about trialling new drugs.  Digital tools may support engagement through life transitions e.g. starting higher education. | The authors call for international collaboration to streamline paediatric MS research, suggesting that one globally coordinated trial per agent would be more efficient than repeating multiple national studies. This would limit unnecessary drug exposure and promote more equitable use of research resources. |
| Paper 39: Weimer et al, Placebo effects in children: a review (66) | | | | |
| Narrative review comparing placebo responses in adults and children across four areas: legal and ethical issues, strength of response, mechanisms, and psychological or contextual influences. | Outlines why placebo responses may be particularly pronounced in children. Regulatory bodies like the FDA and EMA support including children in research. Placebo trials require fewer patients to yield statistically significant results. | Declaration of Helsinki argues against placebo unless no other medications available. | Doctors and parents should be involved in determining the age at which children can appropriately participate in a study. | Compared with adults, legal guidance for inclusion of children in PCTs are not clear and sometimes inconsistent. |
| Paper 40: Wolfe et al, Exploration and ethical analysis of open-label pediatric vaccine trials in a pandemic (67) | | | | |
| Explore the advantages and disadvantages of using open-label vaccine trials for children, particularly for COVID-19, with implications for future pandemics. | Open label study will provide data on tolerability and efficacy but is not as rigorous as a placebo controlled trial. Immune responses can differ in children (e.g. with polysaccharide vaccines). | There are strong reasons to believe the vaccine is not harmful, based on adult data and risk-benefit considerations. | RCTs may be difficult to conduct because parental views about vaccination could limit willingness to enrol, leading to recruitment challenges and biased trial populations. | Certain groups of children may experience greater negative impacts from pandemic measures and may be underrepresented in vaccine research. |
| Paper 41: Kelly et al, Considerations for the design and conduct of pediatric obesity pharmacotherapy clinical trials: Proceedings of expert roundtable meetings (40) | | | | |
| Expert panels (clinicians, regulators, academics, industry, and patient advocates) met in 2022–2023 to develop consensus on key design issues for paediatric anti-obesity trials, including placebo use. | Supports continued use of placebo-controlled trials when scientifically justified (placebo recipients also receive lifestyle therapy which may benefit). Open-label extension phases were recommended to support longer-term safety and efficacy evaluation. | Recognising that placebo use in progressive diseases like obesity may lead to harmful weight gain, the panel advised against extended placebo exposure or post-treatment follow-up without access to therapy. Recommendations included use of active comparators, limiting placebo duration, allowing rescue medication use, and permitting withdrawal if significant weight gain occurs. | The panel stressed the need for transparent, age-appropriate consent and assent procedures. Consent materials should clearly explain that placebo recipients may not benefit from the investigational treatment, supporting informed participation. | Recommended expanding exclusion criteria to include underrepresented groups. They advocated for the inclusion of youth with stable mental health conditions, individuals on stable medications affecting weight, and those with monogenic or syndromic obesity. They also noted that practical barriers, like intensive visit schedules, may limit participation for disadvantaged groups and should be addressed. |
